# Pulmonary Vein Thrombosis in the Setting of COVID-19 Infection: A Case Report

**DOI:** 10.7759/cureus.29539

**Published:** 2022-09-24

**Authors:** Fawaz Araim, Robert J Dabek, Thaer Shehadeh, Michael G Allison

**Affiliations:** 1 General Surgery, Ascension St. Agnes Hospital, Baltimore, USA; 2 Critical Care Medicine, Ascension St. Agnes Hospital, Baltimore, USA

**Keywords:** pulmonary vein thrombosis, prophylaxis, systemic anticoagulation, ards (acute respiratory distress syndrome), covid 19

## Abstract

The novel coronavirus SARS-CoV-2 (COVID-19) affects all three branches of Virchow's triad. It increases the risk of thrombosis and thromboembolic events. Pulmonary embolism and stroke are most commonly reported. However, there is an increasing number of cases demonstrating thrombosis in otherwise uncommon anatomical areas. In this presentation, we will explore the potential causes of pulmonary vein thrombosis secondary to COVID-19.

## Introduction

The novel coronavirus SARS-CoV-2 (COVID-19) has progressed rapidly to reach pandemic levels, killing an estimated nearly one million Americans as of May 8, 2022 [1]. Its presentation varies from asymptomatic to acute respiratory distress syndrome (ARDS) and death. A striking feature of COVID-19 is that it affects every organ system, having various symptoms and complications unrelated to the respiratory system. Notably, patients with COVID-19 have an increased tendency to a prothrombotic state, most commonly presenting as stroke or pulmonary embolism. However, atypical sites of thrombosis have been reported, including basilic vein thrombosis, digital ischemia, and thoracic aorta [2-4]. We present a patient with COVID-19 ARDS who developed pulmonary vein thrombosis (PVT), a rare but life-threatening condition. To our knowledge, only two other cases have been reported of PVT in the setting of COVID-19 pneumonia [4,5].

## Case presentation

**Figure 1:**
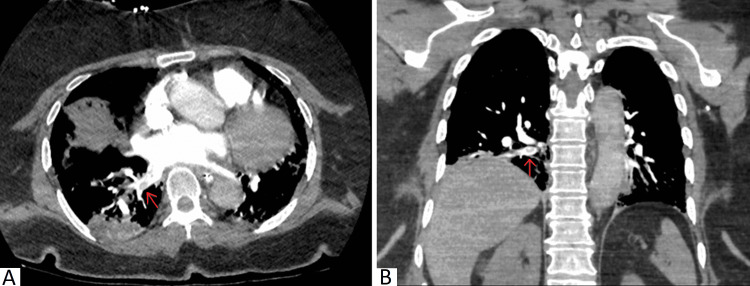
Axial (A) and coronal (B) images on CT angiography demonstrating a filling defect in the right lower lobe pulmonary vein, consistent with pulmonary vein thrombosis.

A 67-year-old African American female with a history of hypertension, hyperlipidemia, and osteoarthritis presented to the emergency department with complaints of shortness of breath and a positive COVID-19 RT-PCR (reverse transcription-polymerase chain reaction) test on admission. Three days prior to admission, she experienced general fatigue, anorexia, productive cough, and dyspnea on exertion. On the day of admission, she experienced shortness of breath, which worsened throughout the day, prompting her to seek medical attention. She arrived at the emergency department via EMS (emergency medical service), and on arrival, she was hypoxic with oxygen saturation at 78% on a 100% oxygen non-rebreather mask. She transitioned to high-flow nasal cannula and eventually reached maximum settings with oxygen saturation ranging between 85% and 95%. She was placed on non-invasive positive pressure ventilation. However, she remained hypoxic, and was eventually intubated five hours after admission. The patient was admitted to the intensive care unit with the diagnosis of ARDS secondary to COVID-19 pneumonia and was placed on dexamethasone and remdesivir for treatment. She was also given subcutaneous heparin for prevention of deep venous thrombosis (DVT). She failed spontaneous awakening trials on the fourth, fifth, and sixth days post-admission. On the seventh day, she developed a fever and began treatment with vancomycin for *Staphylococcus pneumonia*. The patient was extubated on day 14 and placed on high-flow nasal cannula. Due to recurrent desaturations, she was reintubated the following day. A computed tomography (CT) angiography of the chest on day 15 showed a PVT, which was not seen on prior chest CT angiography from day 1 (Figure 1). She began treatment with therapeutic dosing of apixaban. As the patient failed extubation, she required tracheostomy and percutaneous endoscopic gastrostomy (PEG). She was discharged to a rehabilitation facility 20 days after admission and continued her anticoagulation therapy.

## Discussion

The pathophysiology of COVID-19 coagulopathy is poorly understood. Nevertheless, we know that COVID-19 affects all divisions of Virchow's triad: endothelial injury, hypercoagulability, and stasis. Direct cell injury occurs when COVID-19 enters endothelial cells via the angiotensin-converting enzyme 2 (ACE-2) receptor. As COVID-19 invades endothelial cells, it induces inflammation, which activates the complement system, enhancing cellular dysfunction. Furthermore, the systemic inflammatory response and neutrophil extracellular traps (NETs) promote endothelial dysfunction, furthering the prothrombotic state [6,7]. Reportedly, alterations in prothrombotic factor levels occur in severely ill COVID-19 patients. One study found that 15 critically ill patients with COVID-19 pneumonia had plasma viscosity exceeding 90% of normal [8]. Finally, immobilization due to sedation or severe disease causes blood stasis.

PVT is a rare type of DVT that is potentially fatal. It is commonly associated with lung cancer, lung transplants, left atrial thrombus, and pulmonary lobectomy. In addition, polycythemia vera and blunt chest trauma are rare, documented causes of PVT. Previous to the COVID-19 pandemic, infection was not a known etiology for PVT [6,9-11].

The mechanisms that promote PVT development include mechanical, vascular torsion, hypercoagulation, and direct injury. Of these mechanisms, COVID-19 may influence the latter two. PVT may be underdiagnosed as it can be asymptomatic or have nonspecific symptoms such as cough, dyspnea, pleuritic chest pain, and hemoptysis. Furthermore, PVT and COVID-19 pneumonia share the same nonspecific symptoms, and infection with COVID-19 may mask the presence of a PVT [12]. The significance of PVT in the setting of COVID-19 infection can be related to shared complications of pulmonary infarction, pulmonary edema, right ventricular failure, and potential risk of thromboembolic disease [10]. Diagnosis of PVT is through imaging and pathology. The imaging modalities used include transesophageal echocardiography, CT scanning, MRI (magnetic resonance imaging), and pulmonary angiography [4]. There is no specific treatment for PVT. However, anticoagulation is used for preventing thrombus expansion and embolization, and for promoting vein recanalization. It is also important to initiate early DVT prophylaxis. Trending D-dimers and early hypercoagulable workup may also be recommended in patients with a high risk of clot formation.

## Conclusions

COVID-19 infection causes an increased risk of thrombosis due to endothelial dysfunction or hypercoagulability. The rarity and nonspecific presentation of PVT may cause clinicians to overlook it. The concurrence of the PVT in the setting of COVID-19 can potentially increase symptom severity, morbidity, and mortality, but further investigations should be made, and high clinical suspicion is required to establish early diagnosis and treatment.
